# Editorial: Biosurfactants: New Insights in Their Biosynthesis, Production and Applications

**DOI:** 10.3389/fbioe.2021.769899

**Published:** 2021-10-05

**Authors:** Gloria Soberón-Chávez, Rudolf Hausmann, Eric Déziel

**Affiliations:** ^1^ Departamento de Biología Molecular y Biotecnología, Instituto de Investigaciones Biomédicas, Universidad Nacional Autónoma de México, Ciudad Universitaria, Mexico City, Mexico; ^2^ Department of Bioprocess Engineering, Institute of Food Science and Biotechnology, University of Hohenheim, Stuttgart, Germany; ^3^ Centre Armand-Frappier Santé Biotechnologie, Institut National de la Recherche Scientifique (INRS), Laval, QC, Canada

**Keywords:** biosurfactants, oil-industry, soil remediation, bioprocess, glycolipids, lipopeptides

## Presentation

The surface-active properties of all the chemically diverse molecules that are defined as surfactants are essential for a wide variety of industrial applications, such as soaps and detergents, food products, cosmetics, agriculture, oil recovery and bioremediation. A small share of the surfactant market is comprised of biosurfactants (surfactants produced by different organisms, but we will focus on those produced by microorganisms), even though they have clear advantages over chemically synthesized surfactants in terms of their biodegradability, low toxicity and sustainability. This is mainly due to the lower production cost of chemically-produced surfactants and to important technical challenges in the large-scale production of biosurfactants, such as foaming during the fermentation process that are difficult to manage and cause biomass loss from the bioreactor. The aim of this Research Topic “*Biosurfactants: New Insights in Their Synthesis, Production and Applications*” is to present an overview of different perspective and the latest research innovations coming from diverse scientific disciplines that address these fascinating molecules through a multidisciplinary approach. Thus, it comprises different approaches on the synthesis (including metabolic engineering strategies), production (fermentation and downstream processing) and applications of several types of biosurfactants. This Research Topic includes 2 in-depth reviews, 4 minireviews and 15 original research articles, presenting a wide panorama of the state-of-the-art in the rapidly growing field of biosurfactants research.

## News in Biosurfactants Research

Surfactants are chemically diverse compounds that can reduce interfacial and superficial tensions. These characteristics make them amenable to a wide range of industrial applications ranging from oil-recovery, to agriculture, as active ingredients of soaps and detergents, and in the cosmetics, textiles and food industries, among others. Most of the surfactants currently commercialized are partly or fully chemically synthesized and produced at a very low cost, but some are toxic and recalcitrant compounds. An extreme example of this is represented by fluorinated surfactants, a subgroup of polyfluoroalkyl substances (PFAS), which were introduced in the 1940s and are very effective surfactants. Because of their inert properties, they were originally considered safe and were widely used. Today, however, they are considered as persistent organic pollutants that need to be replaced with environmentally friendly alternatives.

Biosurfactants such as those produced by microorganisms, like bacteria and yeasts, represent an eco-friendly alternative (Soberón-Chavez and Maier, 2010). However, they currently still only represent a small share of the market, mainly because of challenges in their large-scale production and their associated high costs (Henkel et al., 2017). Applications of biosurfactants have been reported in a wide range of fields. For example they have been proposed to be useful in drug delivery and other biomedical applications (Ceresa et al., 2021), as well as in the oil-industry where large quantities are needed (for example see the work by Nikolova and Gutierrez in this Research Topic).

**FIGURE 1:**
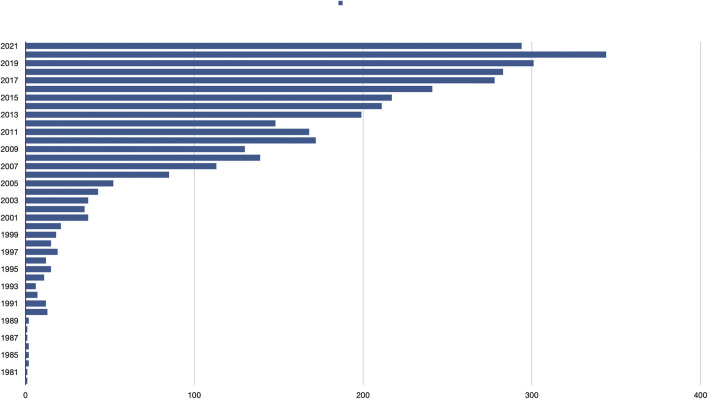
Number of articles reported in PubMed by year on the topic “biosurfactants”.

The biosurfactants research field has grown rapidly since the first publication that used the term « biosurfactant» in 1979; in 2020 344 articles were published and by August of this year there were already nearly 300 publications documented in PubMed (Figure 1). The aim of this research topic was to contribute to this fascinating, multidisciplinary research area including different approaches on the synthesis (including metabolic engineering strategies), production (fermentation and downstream processing) and applications of different types of biosurfactants, in order to present a wide panorama of the state-of-the-art in the field. This objective was fulfilled with the publication of 21 articles that include 2 reviews, 4 minireviews and 15 original research papers. One of the original research papers by Kubicki et al. presents a novel method to quantify different biosurfactants that will be very helpful for the proper detection of these molecules, which is one of the problems that have been identified in the process for the rigorous characterization of biosurfactants and the microorganism that produce them (Twigg et al., 2021).

Even though there are several chemical types of biosurfactants (Nikolova and Gutierrez) research has focused mainly on the study of glycolipids and lipopeptides. The case of lipopeptides produced by various *Bacillus* spp. is systematically reviewed in this Research Topic by Théatre et al. who present the synthesis by non-ribosomal peptide synthetases of the diverse peptides that can be part of these remarkably diverse molecules and how there are 15 lipid isomers that can be linked to these peptides giving rise to a wide range of surfactin-like lipopeptides. Furthermore, this review presents different approaches to produce novel lipopeptide derivatives with diverse physicochemical properties, using synthetic biology, metabolic modeling, and engineering; and finally addresses production processes optimization to increase the productivity of this type of biosurfactants.

In addition, this Research Topic contains two original research articles addressing the case of lipopeptide biosurfactants production with a different focus. Hoffman et al. study the production of surfactin by *Bacillus subtilis* under anaerobic conditions to circumvent the problem of foam production during fermentation. While Biniarz et al. report the development of a bioprocess to produce cyclic lipopeptides pseudofactins (PFs) from *Pseudomonas fluorescens* BD5 cultures that can be used for the efficient production and purification of other lipopeptides of *Pseudomonas* and *Bacillus* origin.

In the case of glycolipids, rhamnolipids that are naturally produced by the opportunistic pathogen *Pseudomonas aeruginosa* and by some *Burkholderia* species (Toribio et al., 2010; Dubeau et al., 2009) are one of the biosurfactants that have been more widely studied (Nitschke et al., 2005). In this Research Topic the regulation of rhamnolipids production by the non-pathogenic species *Burkholderia thailandensis* E264 is addressed by Martinez et al. who show that its production is negatively regulated by the transcriptional regulator ScmR, while the production of polyhydroxyalkanoates (PHA) is positively regulated by this protein. The understanding of the genetic regulation of rhamnolipid production in this bacterial species is key for the development of strains with enhanced production of this biosurfactant that will be suitable for its industrial scale production.

One of the problems for the large-scale production of rhamnolipids using *P. aeruginosa* which has the highest production, is that this bacterium is an opportunistic pathogen (Soberón-Chávez et al., 2021). An alternative strategy to circumvent this problem is the heterologous production of this glycolipidic biosurfactant in a surrogate host such as non-pathogenic *Pseudomonas putida* (Wittgens et al., 2011; Beuker et al., 2016). Several of the articles in this Research Topic address different aspects of rhamnolipids production in *P. putida*. Bator et al., Corrigendum, Tiso et al. and Blesken et al. present different strategies to genetically modify *P. putida* strain KT2440 in order to develop a bioprocess with advantages for rhamnolipids production. In the article by Bator et al. a derivative with enhanced ability to grow using ethanol (a solvent that reduces foam production during fermentation) was selected by experimental evolution, this strain has an increased rhamnolipid production in a fed-batch ethanol fermentation and less foam was produced. Tiso et al. developed expression cassettes for stable integration of the rhamnolipid biosynthesis genes into the chromosome under inducible promoters; in addition, they constructed a strain that redirects carbon flow towards rhamnolipids production by deleting genes involved in flagella and PHA synthesis; rhamnolipids production by this genetically modified *P. putida* KT2440 derivative was improved by using a fermentation process in which the foam produced was recycled. The article by Blesken et al. reports the fractionation of foam to separate rhamnolipids and cells from the culture medium; mutants with deletion of genes encoding hydrophobic membrane proteins were constructed to reduce the partitioning of cells to the gas-liquid interphase and it was shown that the biomass enrichment in the foam of a non-motile derivative that does not produce a flagellum is reduced by 46% compared to the reference strain.

Wittgens and Rosenau review the different challenges and opportunities presented by the heterologous production of rhamnolipids, not only in *P. putida*, but also in other bacteria or even in *Saccharomyces cerevisiae*.

Glycolipids produced by yeasts, such as sophorolipids, are produced in relative high yields and are now commercially available. However, there are other glycolipids produced by yeasts and fungi that have important potential applications, but the conditions for their production are still not well defined. In this Research Topic two original articles address this issue; Beck and Zibek describe a defined mineral culture medium for the growth of fungi belonging to the *Ustilaginaceae* family, and the subsequent use of rapeseed oil for the production of the biosurfactant mannosylerythritol lipid (MEL), while Oraby et al. addressed the production of cellobiose lipid (CL) biosurfactant by the same family of fungi in culture media with different carbon/nitrogen ratio and carbon sources, achieving the production of 17.6 g/L of CL in 1 L bioreactor, producing MEL as a biproduct.

Even though a few microorganisms are well known by their ability to produce biosurfactants, there remains a large diversity of microorganisms to be explored for their ability to produce new molecules. The evaluation of novel biosurfactant producers is address from different points of view in this Research Topic. For example, a minireview by Biniarz et al. presents the potential use as biosurfactant producers of the marine bacteria belonging to the genus *Planococcus* and highlight the importance of exploring marine microorganisms for bioactive molecules that have a potential for industrial use. Subsanguan et al. in an original research article used the immobilized lactic acid bacteria *Weissella cibaria* to produce a glycolipid biosurfactant that has a great potential for its application in products, such as food-grade emulsifiers and cosmetics, among others. Another original research article by Galdino Ribeiro et al. report a novel biosurfactant produced by a traditional food-used microorganism, *Saccharomyces cerevisiae*. They provide evidence for the use of this biosurfactant produced by *S. cerevisiae* URM 6670 as a substitute for egg yolk in the elaboration of cookies. Aráujo et al. analyzed the effect of using mineral medium with petroleum in the culture enrichment of bacteria that are potential biosurfactants producers (evaluated by the presence of genes related to their production) using a production water sample from a Brazilian oil reservoir comparing this medium with the enrichment using yeast extract peptone dextrose (YPD)-rich medium; they concluded that the diversity of the microorganisms enriched is diminished when mineral oil with petroleum is used (predominating *Brevibacillus* genus members), but the metagenomic analysis revealed an increased presence of genes related to biosurfactant production under these selective conditions. *Brevibacillus* is a spore-forming Gram-positive bacterium with high agroecology significance as a potential plant growth-promoting rhizobacterium, biocontrol agent against plant diseases, and is effective in soil bioremediation to remove toxic heavy metals from soils, water, and the atmosphere (Ray et al., 2020).

Among the many applications of biosurfactants, this Research Topic contains articles that address three main areas:

• Nikolova and Gutierrez present a general overview of the different types of microbially-produced biosurfactants and their applications and present the current state of research trends in the use of biosurfactants by the Oil and Gas industry for enhancing oil recovery from exhausted oil fields and as dispersants for combatting oil spills.
• Mulligan presents a minireview addressing the sustainable use of biosurfactants in the remediation of contaminated soils, highlighting the importance of biosurfactant selection which should be based on the properties of the pollutant, treatment capacity, costs, and regulatory requirements; when possible it is advisable to use renewable or waste substrates for biosurfactant production, ideally *in situ*, and to develop a recovery processes for reuse of these compounds. Related to this application of biosurfactants is the original research article by Zhu et al. that refers to the use of a lipopeptide biosurfactant produced by *B. subtilis* N3-1P cultivated in a medium containing fish-waste produced peptone as a dispersant of oil spills, and highlight that it is a cost-efficient process. Arpornpong et al. also addressed the use of biosurfactants in the treatment of soil pollution by total petroleum hydrocarbons (TPHs) which are a major pollutant; this study reports the use of a washing technology prior to bioremediation with a mixture of microorganisms (*Marinobacter salsuginis* RK5, *Microbacterium saccharophilum* RK15 and *Gordonia amicalis* JC11); the washing fluid used contained a mixture of chemical surfactants with the culture broth of a *B. subtilis* strain that produced a lipopeptide; the washing step considerably reduced TPH concentrations and after the bioremediation step with the addition of a fertilizer an efficient process of *in situ* cleaning of soil was reported.
• Crouzet et al. present a minireview addressing the role of lipopeptide and rhamnolipid biosurfactants as plant protection agents, since these tensio-active compounds have a direct antimicrobial activity against plant pathogens, and also provide information on how rhamnolipids and lipopeptides stimulate the plant immune system thus contributing to plant resistance to phytopathogens. The original research article by Chopra et al. study the case of a *P. aeruginosa* strain RTE4 a potential plant promoting rhizobacteria which was isolated from the rhizosphere of a tea plant, showing that it produces di-rhamnolipid (a rhamnolipid molecule with two rhamnose moieties) that possess antifungal activity.

In summary, this Research Topic represents a diverse overview but also provides some detailed insights into current biosurfactants research, even not being exhaustive. Therefore, the readers will acquire a wide view of the state of the art in this promising biotechnological area.
